# Sudapyridine (WX-081) antibacterial activity against *Mycobacterium avium*, *Mycobacterium abscessus*, and *Mycobacterium chelonae in vitro* and *in vivo*

**DOI:** 10.1128/msphere.00518-23

**Published:** 2024-01-19

**Authors:** Luyao Zheng, Hong Wang, Xueting Qi, Weiyan Zhang, Bin Wang, Lei Fu, Xi Chen, Xiaoyou Chen, Yu Lu

**Affiliations:** 1 Department of Pharmacology, Beijing Key Laboratory of Drug Resistance Tuberculosis Research, Beijing Chest Hospital, Capital Medical University, Beijing Tuberculosis and Thoracic Tumor Research Institute, Beijing, China; 2 Tuberculosis Department, Beijing Chest Hospital, Capital Medical University, Beijing Tuberculosis and Thoracic Tumor Research Institute, Beijing, China; 3 Infectious Diseases Department, Beijing Ditan Hospital, Capital Medical University, Beijing, China; Antimicrobial Development Specialists, LLC, Nyack, New York, USA

**Keywords:** non-tuberculous mycobacteria (NTM), sudapyridine (WX-081), J774A.1 macrophages, BALB/c mice

## Abstract

Sudapyridine (WX-081) is a structural analog of bedaquiline (BDQ), which shows anti-tuberculosis and non-tuberculous mycobacteria (NTM) activities but, unlike BDQ, did not prolong QT interval in animal model studies. This study evaluated the antibacterial activity of this novel compound against *Mycobacterium avium*, *Mycobacterium abscessus*, and *Mycobacterium chelonae in vitro* and *in vivo*. The minimum inhibitory concentration (MIC) of WX-081 against three kinds of non-tuberculous mycobacteria (NTM) clinical strains was determined using microplate-based alamarBlue assay (MABA), and the antibacterial activity of WX-081 against NTM in J774A.1 cells and mice was evaluated. MIC ranges of WX-081 against clinical strains of *M. avium* and *M. abscessus* were 0.05–0.94 μg/mL, 0.88–7.22 μg/mL (*M. abscessus* subsp. *abscessus*), and 0.22–8.67 μg/mL (*M. abscessus* subsp. *massiliense*), respectively, which were slightly higher than those of BDQ. For *M. avium*, *M. abscessus*, and *M. chelonae*, WX-081 can reduce the intracellular bacterial load by 0.13–1.18, 0.18–1.50, and 0.17–1.03 log_10_ colony forming units (CFU)/mL, respectively, in a concentration-dependent manner. WX-081 has bactericidal activity against three NTM species in mice. WX-081 exhibited anti-NTM activity to the same extent as BDQ both *in vivo* and *in vitro*. WX-081 is a promising clinical candidate and should be studied further in clinical trials.

**IMPORTANCE:**

Due to the rapidly increased cases globally, non-tuberculous mycobacteria (NTM) disease has become a significant public health problem. NTM accounted for 11.57% of all mycobacterial isolates in China, with a high detection rate of *Mycobacterium abscessus*, *Mycobacterium avium*, and *Mycobacterium chelonae* during 2000–2019. Treatment of NTM infection is often challenging, as natural resistance to most antibiotics is quite common among different NTM species. Hence, identifying highly active anti-NTM agents is a priority for potent regimen establishment. The pursuit of new drugs to treat multidrug-resistant tuberculosis may also identify some agents with strong activity against NTM. Sudapyridine (WX-081) is a structural analog of bedaquiline (BDQ), which was developed to retain the anti-tuberculosis efficacy but eliminates the severe side effects of BDQ. This study initially evaluated the antimicrobial activity of this novel compound against *M. avium*, *M. abscessus*, and *M. chelonae in vitro*, in macrophages and mice, respectively.

## INTRODUCTION

Non-tuberculous mycobacteria (NTM) accounted for 11.57% of all mycobacterial isolates in China, with the highest detection rate of *Mycobacterium abscessus* during 2000–2019 (1). In addition,the detection rates of *Mycobacterium avium* and *Mycobacterium chelonae* were high. Because of the lack of effective drugs, infection with NTM is more difficult to treat than tuberculosis. NTM disease has the characteristics of a long treatment period, multidrug combination therapy, and limited active drugs, which suggests that we need to supplement the anti-NTM drug pipeline as soon as possible.

It has been reported that bedaquiline (BDQ) had good activity against NTM *in vitro* (2). Although BDQ is not explicitly listed in the guidelines as a treatment for NTM disease, it has been successfully used clinically in the empiric therapy of *M. avium*, *M. abscessus*, and *Mycobacterium fortuitum* infections (3–5). Sudapyridine (WX-081) is a new compound obtained by reasonable structural modification and optimization of BDQ. It retains the pharmacological scaffold of BDQ (composed of a quinoline heterocyclic nucleus and side chains of tertiary alcohol and tertiary amine groups), replacing the quinoline group with the pyridine group (6). Compared with BDQ, WX-081 had a higher exposure to target organs in the lung after oral administration and did not prolong the QT interval in animal model studies (6). *In vitro*, the antibacterial activity of WX-081 was comparable to that of BDQ against both susceptible and resistant *Mycobacterium tuberculosis* and different NTMs (7), and WX-081 and BDQ had similar efficacy in acute and chronic mouse tuberculosis models infected with small doses of aerosol (6).

Therefore, we evaluated the antibacterial activity of WX-081 against *M. avium*, *M. abscessus*, and *M. chelonae in vitro*, in macrophages and, firstly, in mice, respectively, in order to provide new drug candidates for the treatment of NTM disease.

## RESULTS

### *In vitro* antimycobacterial activity

The *in vitro* antibacterial activity of WX-081 and BDQ against 31 clinical strains of NTM (9 strains of *M. avium*, 13 strains of *M. abscessus* subsp. *abscessus,* and 9 strains of *M. abscessus* subsp. *massiliense*) was tested by microplate-based alamarBlue assay (MABA). The experiment was repeated three times. The minimum inhibitory concentration (MIC) of these drugs against the corresponding NTM standard strains was determined simultaneously in each experiment as a control.

As shown in Tables 1 and 2, BDQ and WX-081 showed good antibacterial activity against *M. avium* and *M. abscessus* clinical strains *in vitro*. The MIC ranges of the two compounds against clinical strains of *M. avium* were 0.03–0.47 μg/mL and 0.05–0.94 μg/mL, respectively, and against *M. abscessus* subsp. *abscessus* were 0.41–3.82 μg/mL and 0.88–7.22 μg/mL, and against *M. abscessus* subsp. *massiliense* were 0.09–3.21 μg/mL and 0.22–8.67 μg/mL, respectively.

**TABLE 1:** MICs of BDQ and WX-081 against clinical strains of *M. avium^a^*

Mycobacterial species		MIC (μg/mL)	
		BDQ	WX-081
*M. avium*	Reference	0.21	0.38
	2-8	0.04	0.09
	2-9	0.05	0.05
	2-11	0.16	0.06
	2-15	0.03	0.17
	2-16	0.07	0.12
	8-1	0.11	0.23
	8-9	0.47	0.94
	8-11	0.03	0.08
	8-15	0.03	0.09

^*a*^ Bedaquiline (BDQ), Sudapyridine (WX-081), MIC (minimum inhibitory concentrations).

**TABLE 2:** MICs of BDQ and WX-081 against clinical strains of *M. abscessus^a^*

Mycobacterial species		MIC (μg/mL)	
		BDQ	WX-081
*M. abscessus* subsp. *abscessus*	Reference	0.46	3.37
	0021	0.41	0.88
	0023	0.46	0.96
	M-111	3.11	6.25
	M-124	2.84	6.33
	M-163	0.90	3.27
	M-165	1.94	4.03
	M-168	3.82	7.22
	M-172	0.95	2.62
	M-237	3.80	7.08
	M-253	1.22	2.84
	M-269	2.78	5.10
	M-288	1.84	4.08
	M-323	1.74	4.36
*M. abscessus* subsp. *massiliense*	0019	0.09	0.22
	0032	0.10	0.31
	M-119	0.57	1.96
	M-126	3.21	6.84
	M-131	2.59	6.16
	M-133	2.34	5.02
	M-234	1.31	3.02
	M-242	3.27	8.67
	M-274	0.61	1.60

^*a*^ The experiment was repeated three times. MICs are the mean of three experiments.

### Intracellular antimycobacterial activity

To evaluate the antibacterial activity of WX-081 and BDQ against intracellular NTMs in J774A.1 cells, the optimal multiplicity of infection (MOI) and infection time of *M. abscessus* to J774A.1 cells were determined. *M. abscessus* bacterial solution (diluted with Dulbecco’s modified Eagle medium [DMEM] medium containing 10% fetal bovine serum [FBS]) was added to J774A.1 cells at MOI of 1, 3, and 5, respectively. After co-incubation for 6, 12, and 24 hours, the cell growth status was observed, and then the cells were lysed to count the colony forming units (CFU) of intracellular mycobacteria. The experiment was repeated twice. The results are shown in Table 3.

**TABLE 3:** Results of determination of optimal MOI and infection time of J774A.1 cells infected with *M. abscessus* (*n* = 6)^*a*^

Mycobacterial species	MOI	Infection time (hours)	CFU count (log_10_ CFU/mL，x̄ ± *s*）
*M. abscessus*	1	6	6.31 ± 0.06
		12	6.28 ± 0.11
		24	6.12 ± 0.08
	3	6	6.49 ± 0.09
		12	6.37 ± 0.13
		24	6.20 ± 0.10
	5	6	6.76 ± 0.07
		12	6.58 ± 0.06
		24	6.39 ± 0.11

^*a*^ The experiment was repeated two times. The CFU count is the mean ± standard deviation of the results of six experimental holes (*n* = 6).

The results showed that all combinations of MOI and infection time allowed *M. abscessus* to infect macrophages to a great extent. However, J774A.1 cells were observed to aggregate and lose refraction at 24 hours after infection, and some cells were suspended in the culture medium. Aggregated macrophages have insufficient adherent capacity, and washing with 1× phosphate buffer saline(PBS) prior to lysis may cause them to fall off the plate, ultimately resulting in CFU counts less than the cells infected for 12 or 6 hours. After 12 hours of infection, no macrophages died, but some cells gathered into clusters, and the bacterial count was slightly lower than that of cells infected for 6 hours. The J774A.1 cells infected for 6 hours grew well, all cells did not form clumps or die, and the intracellular bacteria could reach more than 6 log_10_ CFU/mL. After 6 hours post-infection, at MOI of 1, 3, and 5, while there was no cell death or aggregation, higher MOI resulted in increased susceptibility to cell damage, thereby affecting the evaluation of intracellular antibacterial activity of BDQ and WX-081. To keep the vector cells in an optimal state to cope with the toxicity of the compound, we finally determined that the optimal MOI for *M. abscessus* infection of the J774A.1 cells was 1, and the optimal infection time was 6 hours.

However, the intracellular bacterial load was only ~4 log_10_ CFU/mL 6 hours after infection with *M. avium* with MOI = 1 (data not shown). Such results demonstrated that the optimal MOI for different NTM-infected macrophages was different. In this study, the J774A.1 cells were infected with *M. avium* and *M. chelonae*, respectively. The macrophages cultured in 24-well plates were added to mycobacteria solution with MOI 1, 3, and 5, respectively. The cell growth was observed, and the number of mycobacteria in the cells was counted 6 hours after infection. The experiment was repeated twice, and the results are shown in Table 4.

**TABLE 4:** Results of determination of optimal MOI of J774A.1 cells infected with *M. avium* and *M. chelonae* (*n* = 6)

Mycobacterial species	MOI	CFU count (log_10_ CFU/mL，x̄ ± *s*）
*M. avium*	1	5.87 ± 0.05
	3	6.63 ± 0.10
	5	6.54 ± 0.07
*M. chelonae*	1	6.24 ± 0.08
	3	6.95 ± 0.04
	5	6.74 ± 0.02

The results showed that after 6 hours of incubation, both *M. avium* and *M. chelonae* could enter macrophages in a large proportion. At 6 hours after infection, the intracellular bacterial counts of *M. avium* were 5.87 ± 0.05, 6.63 ± 0.10, and 6.54 ± 0.07 log_10_ CFU/mL at MOI of 1, 3, and 5, respectively, whereas the intracellular bacterial counts of *M. chelonae* were 6.24 ± 0.08, 6.95 ± 0.04, and 6.74 ± 0.02 log_10_CFU/mL at MOI of 1, 3, and 5, respectively. Therefore, it can be preliminarily determined from the results that the optimal MOI of *M. avium* was 3, and the optimal MOI of *M. chelonae* was 1.

**Fig 1:**
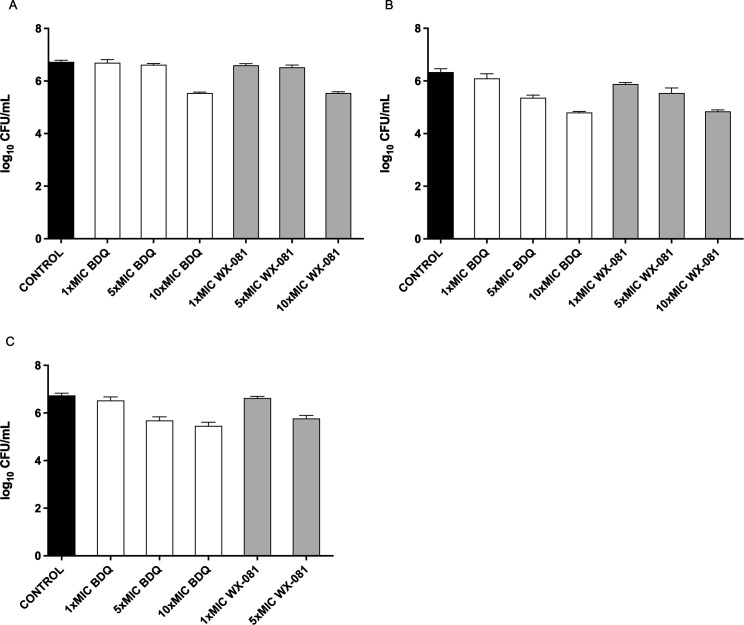
Results of antibacterial activity determination of BDQ and WX-081 against *M. avium* (**A**), *M. abscessus* (**B**), and *M. chelonae* (**C**) in J774A.1 cells

Therefore, under the conditions of optimal MOI and optimal infection time, the antibacterial activities of BDQ and WX-081 against *M. avium* and *M. chelonae* in the J774A.1 cells were tested at 1× MIC, 5× MIC, and 10× MIC, respectively. Since the concentration of WX-081 at 10× MIC for *M. chelonae* was greater than the 50% inhibitory concentration (IC_50_) for J774A.1 cell, the intracellular antibacterial activity of WX-081 at this concentration was not determined. The experiment was repeated twice, and the specific results are shown in Table 5 and Fig. 1.

**TABLE 5:** Antibacterial activity of WX-081 against NTM in J774A.1 cells (*n* = 6)

Mycobacterial species	Concentrations of the compounds	CFU count (log_10_CFU/mL，x̄ ± *s*）		
		Control	BDQ	WX-081
*M. avium*	1× MIC	6.73 ± 0.06	6.70 ± 0.11	6.60 ± 0.07
	5× MIC		6.62 ± 0.05	6.53 ± 0.09
	10× MIC		5.54 ± 0.02	5.55 ± 0.06
*M. abscessus*	1× MIC	6.34 ± 0.13	6.23 ± 0.08	6.16 ± 0.28
	5× MIC		5.47 ± 0.04	5.54 ± 0.18
	10× MIC		4.80 ± 0.05	4.84 ± 0.06
*M. chelonae*	1× MIC	6.20 ± 0.09	6.53 ± 0.14	6.63 ± 0.07
	5× MIC		5.68 ± 0.16	5.77 ± 0.12
	10× MIC		5.46 ± 0.15	—

BDQ and WX-081 showed similar antibacterial activity against mycobacteria within the J774A.1 cells. BDQ reduced intracellular bacterial load by 0.03–1.19, 0.11–1.54, and 0.27–1.34 log_10_ CFU/mL for *M. avium*, *M. abscessus*, and *M. chelonae*, respectively. WX-081 reduced the intracellular bacterial load by 0.13–1.18, 0.18–1.50, and 0.17–1.03 log_10_ CFU/mL, respectively. The activities of the two compounds were concentration dependent.

### *In vivo* efficacy in mice

**Fig 2:**
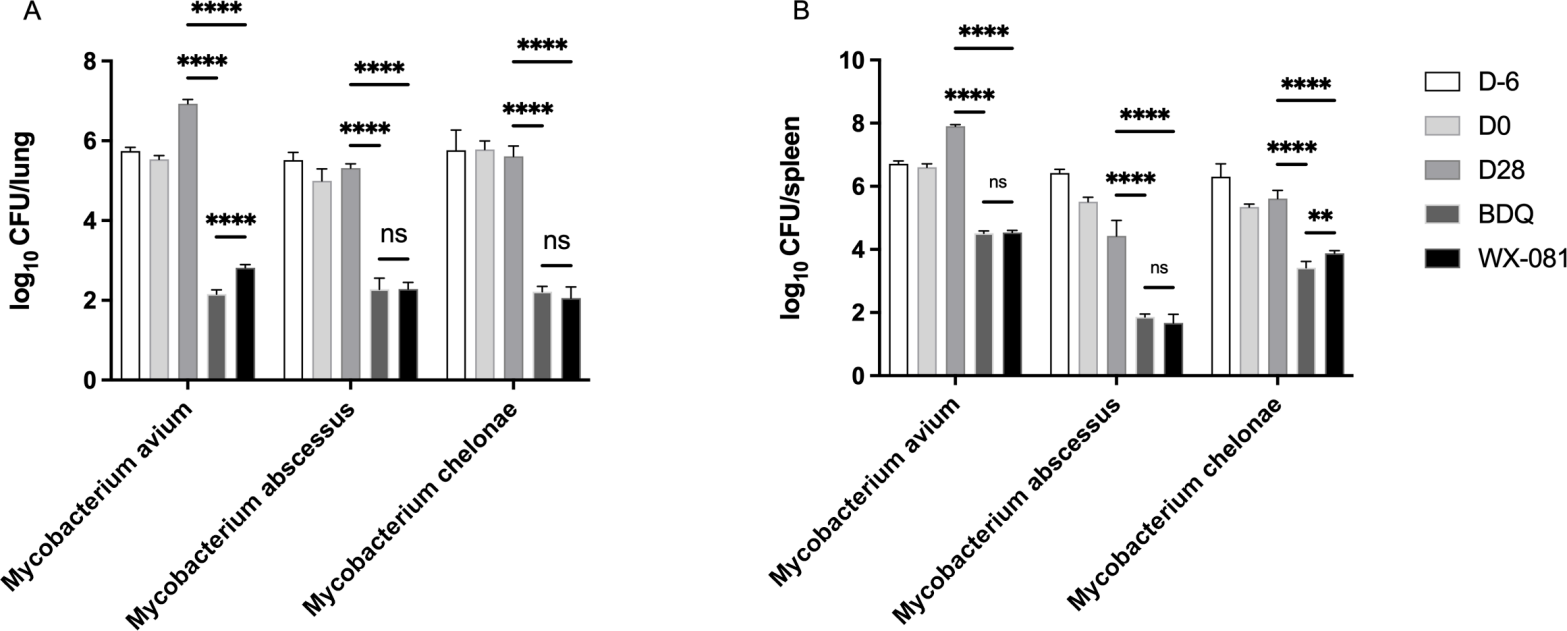
Mean number (log_10_) of CFU per lung (**A**) and spleen (**B**) in various groups of mice infected with *M. avium*, *M. abscessus*, and *M. chelonae*. D-6, 1 day post-infection and 6 days before drug treatment starts; D0, the day when the drug treatment starts; D28, the day when the therapy ends. “D-6, D0, and D28” represent the blank control group, and “BDQ and WX-081” represent the 28-day treatment with BDQ and WX-081 (25 mg/kg). **P < 0.01, ****P < 0.0001; ns, not significant.

Our previous experiments showed that the administration of dexamethasone to mice before and after infection increased the burden of mycobacteria in the organs of mice and made the effect of antibiotics more pronounced (8). So, in this study, we used the same method to establish the mouse models of *M. avium*, *M. abscessus*, and *M. chelonae* infection, and investigated the anti-NTM activity of BDQ and WX-081 *in vivo* (Fig. 2).

After 4 weeks of treatment, there were no differences in lung index and spleen index between the BDQ and WX-081 groups (Table 6). The results showed that BDQ and WX-081 showed similar bactericidal activity against *M. avium*, *M. abscessus*, and *M. chelonae* in mice after 4 weeks of monotherapy; that is, they could reduce the organ bacterial load of mice below the initial bacterial load (D0). The two compounds reduced the *M. avium* load in the lungs of mice by 4.76 and 4.11 log_10_ CFU and by 3.40 and 3.37 log_10_CFU in the spleen, respectively. BDQ was more active in the lungs than WX-081 (*P* < 0.0001). For *M. abscessus*, both compounds reduced the number of lung bacteria by about 3.1 log_10_CFU and the number of spleen bacteria by about 2.6 log_10_ CFU. For *M. chelonae*, they reduced the bacterial load in the lungs of mice by 3.40 and 3.57 log_10_ CFU, respectively; BDQ reduced the bacterial count in the spleen by 2.07 log_10_ CFU and was more active than WX-081 (*P* < 0.01).

**TABLE 6:** Weight and organ index of mice infected with NTMs after drug treatment^*a*^

Mycobacterial species	Organ indices	D28	BDQ	WX-081
*M. avium*	Mean lung index ± SD	0.83 ± 0.06	0.68 ± 0.05	0.70 ± 0.04
	Mean spleen index ± SD	1.36 ± 0.27	1.24 ± 0.08	1.21 ± 0.25
*M. abscessus*	Mean lung index ± SD	1.14 ± 0.29	0.93 ± 0.17	0.85 ± 0.07
	Mean spleen index ± SD	1.36 ± 0.17	1.50 ± 0.06	1.39 ± 0.47
*M. chelonae*	Mean lung index ± SD	0.85 ± 0.14	0.84 ± 0.08	0.82 ± 0.02
	Mean spleen index ± SD	1.00 ± 0.61	1.64 ± 0.24	2.07 ± 0.30

^*a*^ “D28” represents the blank control groups when therapy ends；"BDQ and WX-081" represent the 28-day treatment with BDQ and WX-081 (25 mg/kg).

## DISCUSSION

With the rising incidence of NTM disease worldwide, how to treat NTM lung disease safely and effectively has become the focus of scientists. Because most of the general antibiotics are ineffective, the pulmonary infection caused by NTM is one of the internationally recognized refractory chronic infectious diseases, and it is urgent to find new and effective treatment drugs.

BDQ is a diarylquinoline antibiotic, which can interfere with the proton motive force (PMF) of mycobacteria and inhibit ATP synthase of mycobacteria (9–11). Research has shown that the survival rate of zebrafish embryos infected with *M. abscessus* was significantly higher than that of untreated zebrafish when exposed to the lowest concentration of BDQ (1 µg/mL), which was comparable to that of imipenem 360 µg/mL, and the antibacterial activity was concentration dependent (12). Le Moigne et al. (13) reported that mice given 30 mg/kg BDQ had significantly fewer *M. abscessus* in the lungs and spleen compared with mice given 150 mg/kg amikacin (AMK). In the nonobese diabetic/severe combined immunodeficient (NOD SCID) mouse model, 10 mg/kg of BDQ had similar anti-*M*. *abscessus* activity as 250 mg/kg of clarithromycin (CLR) (14).

In a small clinical trial conducted by Philley et al. (15), 9 of 10 (90%) treatment-unsuccessful patients infected with *M. abscessus* or *M. avium* experienced symptom relief after 2 months of treatment with a BDQ-containing regimen. At 6 months of treatment, sputum culture results improved in 60% of patients. WX-081 has not been used in clinical treatment of NTM-infected patients.

Ruth et al. (2) reported a BDQ MIC ranging from 0.25 to 0.5 µg/mL for rapid-growing mycobacteria (RGM) and 0.03 to 0.25 µg/mL for slow-growing mycobacteria (SGM) in 7H9 liquid medium. Our study also showed that both compounds had good antibacterial activity against clinical strains of *M. avium* and *M. abscessus in vitro*. In addition, BDQ showed excellent bactericidal activity against *M. abscessus* within the human leukemia monocytic cell line THP-1 in a concentration-dependent manner, which was consistent with our experimental results. However, 1× MIC and 5× MIC BDQ or WX-081 had little inhibitory effect on *M. avium* within the J774A.1 cells. Kilinç et al. (16) also reported that BDQ had only partial inhibitory activity against *M. avium* strain 101 at 1.74 µg/mL in primary human macrophages (difference not statistically significant). However, when the concentration of BDQ or WX-081 was increased to 10× MIC, the antibacterial activity of the two compounds against *M. avium* increased significantly. Therefore, BDQ or WX-081 may be a potential drug for the treatment of NTM disease.

As there is no study on the anti-NTM effect of WX-081 in macrophage and mice, our group conducted the first evaluation of the anti-NTM activity of WX-081 at both macrophage and animal levels. In our established mouse model, WX-081 exhibited bactericidal effects on *M. avium*, *M. abscessus* and *M. chelonae*. Therefore, WX-081 and BDQ have similar antibacterial activity against NTMs both *in vitro* and *in vivo*.

Our study also has some limitations. First, the number of clinical strains tested was small, and all of the tested isolates were obtained from Beijing Chest Hospital. Consequently, some isolates could be related epidemiologically. Second, there was a substantial variation in MIC values against *M. abscessus*. Only one reference strain was tested in macrophages and *in vivo*. In our future studies, more species of *M. abscessus* will be used in macrophages and *in vivo* studies. Nonetheless, our study provides a comprehensive description of the antibacterial activity of WX-081 against *M. avium*, *M. abscessus*, and *M. chelonae in vitro* and *in vivo*.

### Conclusion

Sudapyridine (WX-081) effectively inhibited the growth of *M. avium*, *M. abscessus*, and *M. chelonae in vitro* and *in vivo*, and its activity was equivalent to that of BDQ. As such, Sudapyridine (WX-081) represents a potential clinical candidate for incorporation into novel therapeutic anti-NTM regimens.

## MATERIALS AND METHODS

### Compounds

Isoniazid and rifampicin were purchased from Sigma-Aldrich. Dexamethasone and bedaquiline were purchased from Biochempartner Co., Ltd. Sudapyridine (WX-081) was provided by WuXi AppTec (Shanghai) Co., Ltd.

### Strains

SGM strain was *M. avium* (ATCC 25291). RGMs were *M. abscessus* (ATCC 19977) and *M. chelonae* (ATCC 14472). NTMs were obtained from the National Clinical Laboratory on Tuberculosis, Beijing Chest Hospital, and were grown in Middlebrook 7H9 broth (Difco) supplemented with 10% (vol/vol) oleic acid-albumin-dextrose-catalase (OADC) (Becton-Dickinson), 0.2% (vol/vol) glycerol, and 0.05% Tween 80.

### MIC measurements

The MICs of CLR, BDQ, and WX-081 against *M. avium* and *M. abscessus* reference strains and clinical strains (no clinical strains of *M. chelonae* were collected) are tested using the MABA. Specific experimental methods refer to previous studies (17).

### Intracellular activity assay

The J774A.1 cells were grown in cell culture dishes in DMEM containing 10% FBS. The cells were detached with trypsin digestion and were resuspended to a final concentration of 4 × 10^5^ cells/mL. Aliquots (1 mL) of cell suspension were distributed into 24-well plates, and the plates were incubated at 37°C in a 5% CO_2_ incubator for 12 hours (18).

The MOI was the ratio of mycobacterial concentration to cell concentration when NTM was used to infect cells. The best MOI and the best time of infection were defined as the MOI and the time of infection when the cells infected with mycobacterium had the most amount of bacteria and the most complete cell morphology. The NTMs were the reference strains of *M. abscessus*, *M. avium*, and *M. chelonae*, respectively. The culture medium of DMEM (containing 10% FBS) was used to mix the *M. abscessus* in a logarithmic growth period into 4 × 10^5^ CFU/mL (MOI = 1), 1.2 × 10^6^ CFU/mL (MOI = 3), and 2 × 10^6^ CFU/mL (MOI = 5). Infection was carried out for 6, 12, and 24 hours, followed by three times of washing with 1× PBS to remove the extracellular mycobacteria. Monolayers were visually inspected under the microscope to ensure they remained intact, and then the medium was removed and the macrophages were lysed with 200 µL of 0.1% sodium dodecyl sulfate. Then the lysates were diluted with fresh media and plated onto 7H10 plates supplemented with 10% OADC to measure the CFU (19). The optimal MOI values of *M. avium* and *M. chelonae* were determined by the same method after the optimal infection time was determined.

The J774A.1 cells were co-incubated with DMEM medium containing 10% FBS and different concentrations of BDQ and WX-081 (1, 5, and 10 times the MICs of the compounds against the standard strains) for 48 hours (20), and the cells were lysed at 48 hours and CFU counts were performed. Cell lysis and CFU counts were then performed as in our previous study. Control wells received drug-free medium (19).

### Establishment of infection in mice

A total of 75 BALB/c mice were given dexamethasone (5 mg/kg) by gavage from 7 days before infection to 6 days after infection, once a day, 0.2 mL each time, and no drug was given on the day of infection. Normal saline was used to dilute the *M. avium*, *M. abscessus*, and *M. chelonae* bacterial solution at the logarithmic growth stage to 1 × 10^7^ CFU/mL. These 75 mice were randomly divided into four groups and infected with three NTMs by tail vein injection (0.2 mL each) (8), respectively.

Five mice infected with different NTMs were randomly grabbed and sacrificed 1 day after infection (D-6) and on the day of treatment initiation (D0). Serial dilutions of tissue homogenates were inoculated on 7H10 solid medium, and the initial number of mycobacteria implanted in lungs and spleens was determined.

### Experimental chemotherapy trials

Fifteen mice infected with each NTM were randomly divided into three groups. One group was treated with CMC as a control group, and the other two groups were treated with BDQ and WX-081 on day 7 post-infection (D0). Both BDQ and WX-081 were suspended in CMC solution as single agents and were given for 28 days (seven times weekly, 0.2 mL each time) by gavage. The dose of BDQ was 25 mg/kg. Since WX-081 is a structural analog of BDQ and has similar antibacterial activity against NTMs *in vitro* and in macrophages to BDQ, we chose the same dose as BDQ.

### Statistical analysis

Figures were plotted using GraphPad prism 8.0 (GraphPad Inc., USA) software, and the data were statistically analyzed using SPSS 26.0. Measurement data are expressed as mean ± standard deviation (x̄± *s*). Organ CFU counts were log-transformed before analysis, and the mean CFU counts were compared by one-way analysis of variance with Dunnett’s *post hoc* test to control for multiple comparisons. The Mann-Whitney test was used to test for significance on non-normally distributed CFU data. A *P* value of 0.05 was considered significant.
